# Application of Intersectionality Framework andArea-level Indicators in Machine Learning Analysisof Depression Disparities in All of Us ResearchProgram Data

**DOI:** 10.21203/rs.3.rs-5536130/v1

**Published:** 2024-12-03

**Authors:** Dmitry Scherbakov, Michael T. Marrone, Leslie A. Lenert, Alexander V. Alekseyenko

**Affiliations:** Medical University of South Carolina; Medical University of South Carolina; Medical University of South Carolina; Medical University of South Carolina

**Keywords:** Depression, Mental health disparities, Intersectionality, Social Determinants of Health, LASSO, All of Us Research Program, Area-Level Indicators

## Abstract

**Background/objective:**

Depression is a complex mental health disorder influenced by various social determinants of health (SDOH) at individual and community levels. Area-level factors and intersectionality framework, which considers overlapping personal identities, are used in this paper to get a nuanced picture of depression disparities.

**Methods:**

This cross-sectional study uses electronic health records data from the All of Us research network. Our study cohort includes 20,042 individuals who completed the SDOH surveys in All of Us and had at least one in-patient visit, with 27.3% diagnosed with depression since 2020. We used depression diagnosis as an outcome, while independent variables include US Religious Census and American urvey responses, area-level variables, sociodemographic characteristics: age group, income, gender, sexual orientation, immigration status, marital status, and race/ethnicity – and the interactions of the latter with each other and with other variables. The association between depression diagnosis and the variables is reported by fitting the logistic regression model on the subset of variables identified by LASSO method.

**Results:**

The analysis revealed that area-level indicators, such as religious adherence and childbirth rates, significantly influenced depression outcomes when interacting with personal identity variables: area-level religious adherence was associated with increased depression odds for women (OR 1.33, 95% CI 1.15–1.54) and non-binary individuals (OR 3.70, 95% CI 1.03–13.31). Overlapping identities, such as younger adults unemployed for less than a year and never married Middle Eastern and North African participants showed higher depression odds (OR 2.3, 95% CI 1.06–4.99, and OR 3.35, 95% CI 1.19–9.45, respectively).

**Discussion/Conclusion:**

The findings underscore the importance of considering all types of factors: individual, area-level, and intersectional in depression research.

## INTRODUCTION

Depression is a multifaceted mental health disorder influenced by various social determinants of health (SDOH) that encompass individual, community, and societal factors. Research has consistently shown that sociodemographic factors, social support, and adverse life experiences significantly contribute to the prevalence and severity of depressive symptoms across diverse populations [1–3].

Intersectionality theory, which was originally developed to acknowledge the combined effects of sex, gender, race, and ethnicity on life outcomes, [4] can be projected into the domain of human health to show that different intersections of identity, social position, processes of oppression or privilege, and policies or institutional practices contribute to health outcomes. The intersectionality framework provides researchers with a perspective to view individuals’ overlapping identities that interact to shape their experiences, in addition to traditional demographic characteristics [5]. In the mental health gender disparities area, the framework may reveal the well-known difference between females and males in depression prevalence rates [6], among other more intricate underlying inequalities created by social structures. For instance, these structures may favor women homemakers and male breadwinners [7]. Intersectionality methods allow us to understand the nature of mental health disparities by analyzing separately working and non-working men and women, moving away from the more general “females” vs. “males” comparisons.

Fehrenbacher & Patel argue that integrating intersectionality into quantitative and mixed-methods research can help address persistent health inequalities [8]. A study by Persmark et al. emphasizes the importance of contextualizing health issues within an intersectional framework in the context of the U.S. opioid crisis, where individual risk factors often overshadow underlying systemic causes of health disparities [9]. This perspective is echoed by Ogungbe et al., who highlight the need for intersectionality to address implicit biases in healthcare that contribute to disparities [10].

The intersectionality framework has been applied to various health contexts, revealing how specific groups experience compounded disadvantages. For example, a study on disability trajectories illustrates how race, ethnicity, and gender intersect to influence health outcomes, suggesting that health disparities are not uniform but rather contingent upon the specific identities individuals hold [5]. This is further supported by research that examines how sexual orientation and race intersect to affect health risk factors, indicating that individuals with multiple marginalized identities face greater health challenges [11], with specific instruments developed to quantify the intersectional effects, for example, related to discrimination [12].

The practical implementation of intersectionality research in healthcare is hindered by specific modeling challenges – namely, the growth of the number of independent variables as a researcher accounts for the interaction effects of a significant number of demographic and social variables. For example, a model with 10 sociodemographic independent variables, each having only 3 levels, results in 200 binary encoded variables when all possible interactions are considered. The growth of the number of variables leads to issues, such as model not being generalizable when number of observations per each variable in minority class is small [13].

Thus, statistical modeling of intersectionality requires special techniques. This paper explores the LASSO variable selection method [14] in order to reduce the dimensions of the design matrix and highlight the most significant intersectional variables that affect depression. LASSO is an efficient method to subset variables and shows superior performance to stepwise variable selection methods [15].

**Figure 1:**
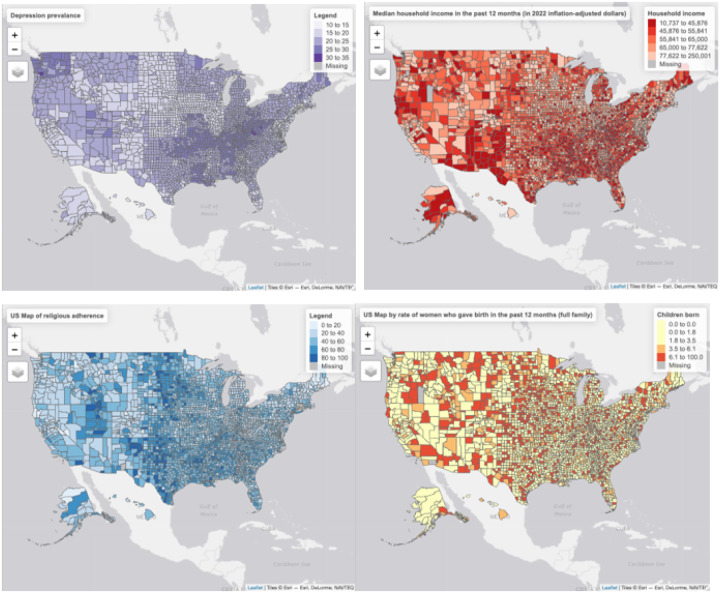
US County-level data for A: Depression prevalence, %; B: Median household income in USD; C: Percentage of women who gave birth in intact family; D: Percentage of religious adherents.

In addition to individual-level SDOH operates at the community level. A secondary goal of this publication is to explore the simultaneous influence of area-level predictors on depression. It is known that lower individual SES is associated with an increased risk of depression due to factors such as financial stress, limited access to healthcare, and reduced educational opportunities [16, 17]. The correlation of household income with depression rates is apparent when two maps are compared side by side (Figs. 1A and 1B), with regions with high depression (e.g., Appalachian) correlating with lower-income regions. At the same time, living in areas where median income is lower than the national average can affect depression through indirect pathways, even if individual or family income is on par with national averages. For example, a middle-class family living in a deprived area might be exposed to higher levels of neighborhood crime and illicit drug exposure, factors associated with lower-income areas [18].

Area-level factors related to income, family structure, percentage of women who give birth, and religious activity are explored in this paper. Positive religious coping is known to provide emotional support and a sense of meaning, which may buffer against depressive symptoms [19, 20]. At the same time, studies highlight that individuals with strong social networks and supportive relationships tend to experience lower levels of depressive symptoms [21, 22]. Differences in family compositions and religious adherence form distinct geographic spots, shaping areas that can be called “family islands” and “religious islands” (Fig. 1C and Fig. 1D).

To the best of our knowledge, prior studies didn’t investigate how these area-level indicators, together with individual-level predictors, and the interaction between predictors influence depression prevalence rates. The present study combines individual-level sociodemographic indicators from The All of Us Research Program and several area-level predictors derived from All of Us 3-digit ZIP code level data, the American Community Survey (ACS), and the 2020 US Religion Census. We aim to identify which of these variables are statistically significant in predicting depression diagnosis in electronic health records (EHR). Individual-level factors such as age, race/ethnicity, depression diagnosis, and other relevant individual data are obtained from the All of Us platform. The All of Us program’s commitment to diversity and inclusion is essential to capture a broad range of intersectional health outcomes in this study [23].

## METHODS

Our general approach follows these steps: 1. Identify individuals in the All of Us dataset diagnosed for depression on or after January 1st, 2020 and those never diagnosed with depression; 2. Link area-level indicators to individual data using a 3-digit ZIP code; 3. Perform LASSO variable selection; 4. Fit a logistic regression model using variables selected by LASSO and plot statistically significant variables.

Identification of people with depression was performed using the All of Us platform phenotyping algorithm, which suggests diagnosis based on the term entered (“depression”). We limited our cohort to patients with at least one in-office visit, who have EHR data present, and who have completed the SDOH survey at any point in time. The SDOH survey contains a range of questions related to social and community context, economic stability, education, neighborhood and built environment, health, and health care [24].

We marked patients as having depression if they had a diagnostic code suggested for the term “depression” (and minimally filtered to exclude unrelated conditions, e.g., myocardial depression, see Table 1) in their EHR, and the diagnosis date was January 1st, 2020, or later. This cut-off date was selected because surveys in All of Us were completed by participants after 2020, and thus, they re ect only recent participant information.

In addition, we considered childbirth (since 2020) as the independent variable and a similar phenotyping process was done to capture deliveries and childbirth.

Area-level indicators are described in Table 2. Because All of Us provides individual participant location using a 3-digit ZIP code, we had to aggregate ACS and US Religion Census data into this higher geographical level using the mean values approach. We also binned all area-level indicators into “Low” (less than one SD from the mean), “High” (higher than one SD from the mean), and “Average” (in between Low and High).

We used these area-level predictors, together with individual predictors from the All of Us platform, and intersectional variables created by specifying variable interactions of a select number of individual predictors known to be correlated with depression prevalence (Age group; Per capita income, calculated as Household income divided by number of people in household; Gender identity; Marital status; Sexual orientation; Race; and Birthplace, In- or outside the US) with each other and with other predictors. The individual, area-level, and variables selected for interaction, corresponding number of participants, and univariable odds ratios are described in Table 3.

The categorical variables were encoded using one-hot encoding into binary predictors. The LASSO regression method is then used for variable selection. We use the logistic regression model on the set of predictors identified by the LASSO to calculate and then present our main result – the impact of area and individual level predictors on depression – through an odds ratio diagram where we display only significant predictors (p-value < .05).

The supplement provides information about the AIC criterion of the regression model, and demonstrates that the model created with LASSO variables provides the best fit compared to the model with all predictors and the model created by fitting only statistically significant predictors separately (Supplemental Table 3).

## RESULTS

The matching All of Us cohort had 20,042 individuals; their demographics, along with individual and area-level variables used in the analysis, are presented in Table 3. In our cohort, 27.3% had a depression diagnosis since 2020.

**Figure 3.**
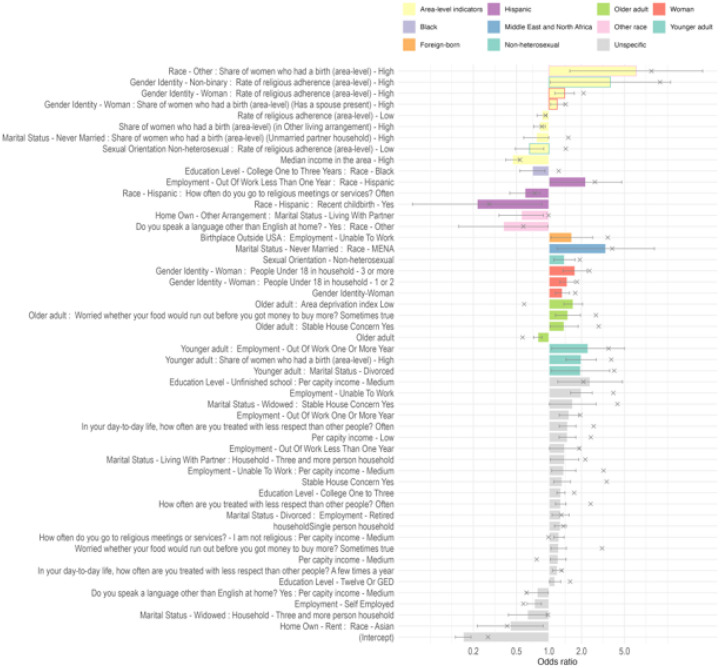
Odds ratio analysis for predictors with p-value < 0.05. Values greater than one indicate a higher chance of occurrence of depression diagnosis. The border around the column indicates the interaction between area and individual variables. 95% CI displayed. “x”-shaped points indicate unadjusted odds ratio (Supplemental Table 1).

The initial list contained 1,003 binary independent variables (that includes 27 categorical variables transformed using one-hot encoding and the specified interactions between them). LASSO method identified 181 important variables. The logistic regression modeling results on the variables selected by LASSO method are displayed in Fig. 3, which shows only the predictors with p-value < .05 (and hence confidence intervals not overlapping with 1 on the odds ratio scale).

The variable interaction with the highest odds was **Race - Other : Share of women who had a birth (area-level) - High** (OR 6.41, 95% CI 1.56–26.38), followed **by Gender Identity - Non-binary : Rate of religious adherence (area-level) - High** (OR 3.70, 95% CI 1.03–13.31). **Marital Status - Never Married : Race - MENA** (OR 3.35, 95% CI 1.19–9.45) and **Education Level - Unfinished school : Per capita income - Medium** (OR 2.40, 95% CI 1.21–4.78) also increased the odds of the outcome. Other significant positive factors included Employment - Unable to Work (OR 1.99, 95% CI 1.58–2.51), and **Younger adult : Share of women who had a birth (area-level) - High** (OR 1.98, 95% CI 1.44–2.74). Notably, Younger adult : Marital Status - Divorced (OR 1.96, 95% CI 1.05–3.65) and **Older adult : Area deprivation index Low** (OR 1.68, 95% CI 1.38–2.04) were also associated with an increase in the outcome.

On the other hand, the variables with the lowest odds included **Race - Hispanic : Recent childbirth - Yes** (OR 0.22, 95% CI 0.05–0.86), additionally, **Do you speak a language other than English at home? - Yes : Race - Other** (OR 0.38, 95% CI 0.15–0.98) showed a negative association, as did **Home Own - Rent : Race - Asian** (OR 0.43, 95% CI 0.22–0.88). **Sexual Orientation - Non-heterosexual : Rate of religious adherence (area-level) - Low** (OR 0.66, 95% CI 0.48–0.89) and Marital Status - Widowed : Household - Three and more person household (OR 0.63, 95% CI 0.42–0.95) were also associated with lower odds. Other variables with negative associations included **Education Level - College One to Three Years : Race - Black** (OR 0.70, 95% CI 0.54–0.91), **Employment - Self Employed** (OR 0.73, 95% CI 0.62–0.85), and **Home Own - Other Arrangement : Marital Status - Living With Partner** (OR 0.55, 95% CI 0.35–0.89).

Supplementary Table 1 provides uni and multi-variate modeling of all predictors selected by LASSO, not just statistically significant, while Supplementary Table 2 provides a similar analysis for base variables without interactions.

## DISCUSSION

Our statistical learning analysis using LASSO and logistic regressions reveals interesting patterns related to intersectionality and interactions with area-level SDOH. Below, we will comment on some of the major findings.

In general, area indicators were comparable in strength and, in some cases, stronger than similar individual-level predictors in our model. For example, individual religious adherence was not a significant predictor in the model, while the interaction of the variable reflecting area religious adherence with gender and sexual orientation was statistically significant. While it is known that women and non-binary individuals face higher rates of depression [25–27], the present study highlights the influence of area factors. Religious organizations can exert pressure on their members, and this can explain the significance of this area predictor.

Similarly, living in an area with a relatively higher number of childbirths was an important predictor of depression for the younger population. In addition, having one, two, or more children in the household was a predictor of depression, but specifically for women, this effect was strong and statistically significant. This finding seems to be confirmed by other studies that suggest that approximately 10% of postpartum women experience major depressive symptoms, with higher rates observed among minority and low-income populations [28, 29].

Belonging to Middle Eastern and North African race and having never married was related in our cohort (which consists mostly of middle-aged and older adults) to higher depression incidence. It is possible that cultural factors related to religious pressures to become married can influence this effect [30].

Unemployment was a strong predictor of depression independent of interaction with demographics, but being out of work for less than one year seemed to be an especially important predictor for the Hispanic subgroup. Similarly, being unemployed was another strong predictor for those born outside of the US. Indeed, research shows that immigrants and historically marginalized groups have limited wealth, and unemployment can lead to quick depletion of resources necessary for sustenance and maintaining the living standard [31, 32]. These specific intersectional effects were much stronger in magnitude than, for example, a simple predictor of having low per capita income.

Some intersectional and interactional effects reduced depression incidence, for example, Asian home renters, individuals of other races who speak a second language at home, and the Hispanic subgroup who had recent childbirth episodes. These effects, while being harder to interpret, were still statistically significant and present an interesting observation.

Another interesting finding is that windowed people living with other people (3 or more people households) seem to have reduced odds of depression. This is echoed by research that shows that older widows living alone are particularly vulnerable to depressive symptoms, suggesting that loneliness plays a critical role in their mental health outcomes [33].

Some individual SDOH variables, although less strong, were still significant in our model, with the most important variables related to per capita income, discrimination, food, and housing insecurity – factors that have long been associated with elevated depression rates [34, 35].

### Limitations

Not all possible variable interactions were explored in this analysis. The initial design matrix had only a curated list of demographic and social variables, interactions for which were explored. Adding a larger number of interactions was possible, but it implied significant computation time in the All of Us platform and was not performed. Although the comparison of magnitudes is difficult because some confidence intervals are too wide, we think that our discussion points are still valid because we can observe the direction of effect for each variable and compare it to previously published results.

### Conclusions

In this work, we fit the LASSO regression to highlight significant area-level and intersectional predictors for depression. This study highlights the importance of environmental and social factors for historically marginalized populations. This study underscores the importance of modeling the overlapping identities through variable interactions when analyzing mental health and other health outcomes in EHR research networks and databases. Our findings also suggest that specific area parameters, such as rate of childbirth and religious adherence (“family” and “religious islands”), have an unexpected influence on some historically marginalized groups.

## Figures and Tables

**Table 1:** Depression phenotype in All of Us platform.

Diagnosis	OMOP ID	SNOMED Code
Acute depression	37016718	712823008
Bipolar affective disorder, current episode depression	439254	191627008
Bipolar disorder, most recent episode depression	35624743	767631007
Bipolar I disorder, most recent episode depression	35624748	767636002
Chronic depression	4103574	192080009
Depression screening positive	762504	428181000124104
Depressive disorder	440383	35489007
Depressive episode	3656234	871840004
Major depression in full remission	4269493	63412003
Major depression in partial remission	4148630	30605009
Major depression in remission	4176002	42810003
Major depression single episode, in partial remission	4323418	70747007
Major depression with psychotic features	37111697	726772006
Major depression, single episode	4282096	36923009
Major depressive disorder	4152280	370143000
Mild depression	4149320	310495003
Mild major depression	4336957	87512008
Mild major depression, single episode	4195572	79298009
Mild recurrent major depression	4228802	40379007
Minimal depression	36713698	718636001
Mixed anxiety and depressive disorder	4338031	231504006
Moderate depression	4151170	310496002
Moderate major depression	4307111	832007
Moderate major depression, single episode	4049623	15639000
Moderate recurrent major depression	4077577	18818009
Moderately severe depression	36717092	719593009
Positive screening for depression on PHQ-9 (Patient Health Questionnaire 9)	43021839	112001000119100
Postpartum depression	4239471	58703003
Presenile dementia with depression	377527	191455000
Psychosis and severe depression co-occurrent and due to bipolar affective disorder	35622934	765176007
Reactive depression (situational)	4314692	87414006
Reactive depressive psychosis	435520	191676002
Recurrent depression	4098302	191616006
Recurrent major depression	4282316	66344007
Recurrent major depression in full remission	4263748	46244001
Recurrent major depression in partial remission	4141454	33135002
Recurrent major depression in remission	433991	68019004
Severe depression	4149321	310497006
Severe major depression	42872722	450714000
Severe major depression with psychotic features	4250023	73867007
Severe major depression without psychotic features	4327337	75084000
Severe major depression, single episode	42872411	251000119105
Severe major depression, single episode, with psychotic features	438406	430852001
Severe major depression, single episode, without psychotic features	441534	76441001
Severe recurrent major depression	43531624	281000119103
Severe recurrent major depression with psychotic features	4154309	28475009
Severe recurrent major depression without psychotic features	435220	36474008
Single episode of major depression in full remission	4025677	19527009

**Table 2:** Area-level indicators.

Indicator	Source	Variable name (in the source)
Median income in the area, US$	All of Us 3-digit ZIP code level data	median_income
Women who gave birth in the past year, %	5-year (2018–2022) ACS	B13004_002
Women who gave birth in the past year, with a spouse present, %	〃	B13004_003
Women who gave birth in the past year, in an unmarried partner household, %	〃	B13004_004
Women who gave birth in the past year, in other living arrangement, %	〃	B13004_005
Share of religious adherents (all denominations/religious groups), %	US Religion Census 2020	TOTRATE_2020

**Table 3:** Sociodemographic profile of study cohort.

Variable		No Depression, N (%)	Depression, N (%)	OR (univariable)
Total N (%)		14564 (72.7)	5478 (27.3)	-
Age groupt	Adults (40 to 69 years old)	6048 (41.5)	2838 (51.8)	-
	Older adults (70 + years old)	7560 (51.9)	2096 (38.3)	0.59 (0.55–0.63, p < 0.001)
	Younger adults (< 40 years old)	956 (6.6)	544 (9.9)	1.21 (1.08–1.36, p = 0.001)
Home Ownership	Own	11316 (77.7)	3440 (62.8)	-
	Other Arrangement	652 (4.5)	363 (6.6)	1.83 (1.60–2.09, p < 0.001)
	Rent	2596 (17.8)	1675 (30.6)	2.12 (1.97–2.28, p < 0.001)
Education Level	College Graduate	4345 (29.8)	1426 (26.0)	-
	Advanced Degree	5819 (40.0)	1570 (28.7)	0.82 (0.76–0.89, p < 0.001)
	College One to Three	3096 (21.3)	1727 (31.5)	1.70 (1.56–1.85, p < 0.001)
	Unfinished school	148 (1.0)	100 (1.8)	2.06 (1.58–2.67, p < 0.001)
	Never Attended	1 (0.0)	2 (0.0)	6.09 (0.58–131.19, p = 0.140)
	Twelve Or GED	1155 (7.9)	653 (11.9)	1.72 (1.54–1.93, p < 0.001)
Sexual Orientation†	Straight	13621 (93.5)	4833 (88.2)	-
	Non-heterosexual	943 (6.5)	645 (11.8)	1.93 (1.73–2.14, p < 0.001)
Do you speak a language other than English at home	No	12898 (88.6)	4916 (89.7)	-
	Yes	1666 (11.4)	562 (10.3)	0.89 (0.80–0.98, p = 0.018)
Marital Status†	Married	9082 (62.4)	2638 (48.2)	-
	Divorced	1872 (12.9)	1089 (19.9)	2.00 (1.84–2.18, p < 0.001)
	Living With Partner	619 (4.3)	302 (5.5)	1.68 (1.45–1.94, p < 0.001)
	Never Married	1768 (12.1)	921 (16.8)	1.79 (1.64–1.96, p < 0.001)
	Separated	163 (1.1)	137 (2.5)	2.89 (2.29–3.64, p < 0.001)
	Widowed	1060 (7.3)	391 (7.1)	1.27 (1.12–1.44, p < 0.001)
Gender Identityt	Man	6096 (41.9)	1557 (28.4)	-
	Non-binary	46 (0.3)	57 (1.0)	4.85 (3.28–7.21, p < 0.001)
	Woman	8422 (57.8)	3864 (70.5)	1.80 (1.68–1.92, p < 0.001)
Birthplace†	USA	13217 (90.8)	5126 (93.6)	-
	Outside USA	1347 (9.2)	352 (6.4)	0.67 (0.60–0.76, p < 0.001)
Within the past 12 months were you worried whether your food would run out before you got money to buy more	Never true	13558 (93.1)	4351 (79.4)	
	Often true	174 (1.2)	261 (4.8)	4.67 (3.85–5.69, p < 0.001)
	Sometimes true	832 (5.7)	866 (15.8)	3.24 (2.93–3.59, p < 0.001)
Living Situation - People Under 18	0	12424 (85.3)	4339 (79.2)	-
	1 or 2	1880 (12.9)	956 (17.5)	1.46 (1.34–1.59, p < 0.001)
	3 or more	260 (1.8)	183 (3.3)	2.02 (1.66–2.44, p < 0.001)
Employment Status	Employed For Wages	5244 (36.0)	1784 (32.6)	-
	Homemaker	379 (2.6)	222 (4.1)	1.72 (1.44–2.05, p < 0.001)
	Out Of Work Less Than One	214 (1.5)	152 (2.8)	2.09 (1.68–2.59, p < 0.001)
	Out Of Work One Or More	324 (2.2)	233 (4.3)	2.11 (1.77–2.52, p < 0.001)
	Retired	6474 (44.5)	1861 (34.0)	0.84 (0.78–0.91, p < 0.001)
	Self Employed	1100 (7.6)	249 (4.5)	0.67 (0.57–0.77, p < 0.001)
	Student	144 (1.0)	85 (1.6)	1.74 (1.32–2.27, p < 0.001)
	Unable To Work	685 (4.7)	892 (16.3)	3.83 (3.42–4.29, p < 0.001)
Stable House Concern	No	13877 (95.3)	4685 (85.5)	-
	Yes	687 (4.7)	793 (14.5)	3.42 (3.07–3.81, p < 0.001)
Race†	White	12190 (83.7)	4551 (83.1)	-
	Asian	305 (2.1)	63 (1.2)	0.55 (0.42–0.72, p < 0.001)
	Black	1035 (7.1)	458 (8.4)	1.19 (1.06–1.33, p = 0.004)
	Hispanic	814 (5.6)	315 (5.8)	1.04 (0.91–1.18, p = 0.601)
	MENA	92 (0.6)	33 (0.6)	0.96 (0.64–1.42, p = 0.844)
	NHPI	10 (0.1)	4 (0.1)	1.07 (0.29–3.20, p = 0.907)
	Other	118 (0.8)	54 (1.0)	1.23 (0.88–1.68, p = 0.218)
Recent childbirth	No	14248 (97.8)	5390 (98.4)	-
	Yes	316 (2.2)	88 (1.6)	0.74 (0.58–0.93, p = 0.012)
Median income in the area	Average	11018 (75.7)	4392 (80.2)	-
	High	2635 (18.1)	585 (10.7)	0.56 (0.51–0.61, p < 0.001)
	Low	911 (6.3)	501 (9.1)	1.38 (1.23–1.55, p < 0.001)
Area deprivation index	Average	9869 (67.8)	4197 (76.6)	-
	High	2690 (18.5)	778 (14.2)	0.68 (0.62–0.74, p < 0.001)
	Low	2005 (13.8)	503 (9.2)	0.59 (0.53–0.65, p < 0.001)
Estimate Total Women who had a birth in the past 12 months (total)	Average	10736 (73.7)	3711 (67.7)	-
	High	1971 (13.5)	1052 (19.2)	1.54 (1.42–1.68, p < 0.001)
	Low	1857 (12.8)	715 (13.1)	1.11 (1.01–1.22, p = 0.024)
Estimate Total Women who had a birth in the past 12 months Has a spouse present	Average	7935 (54.5)	3172 (57.9)	-
	High	2739 (18.8)	1075 (19.6)	0.98 (0.90–1.07, p = 0.660)
	Low	3890 (26.7)	1231 (22.5)	0.79 (0.73–0.85, p < 0.001)
Estimate Total Women who had a birth in the past 12 months Partner in an unmarried partner household	Average	9963 (68.4)	3576 (65.3)	-
	High	2506 (17.2)	1136 (20.7)	1.26 (1.17–1.37, p < 0.001)
	Low	2095 (14.4)	766 (14.0)	1.02 (0.93–1.12, p = 0.691)
Estimate Total Women who had a birth in the past 12 months Other living arrangement	Average	9925 (68.1)	3985 (72.7)	
	High	2727 (18.7)	902 (16.5)	0.82 (0.76–0.90, p < 0.001)
	Low	1912 (13.1)	591 (10.8)	0.77 (0.70–0.85, p < 0.001)
Rate of religious adherence county level	Average	9962 (68.4)	3450 (63.0)	-
	Low	2828 (19.4)	1005 (18.3)	1.03 (0.95–1.11, p = 0.536)
	High	1774 (12.2)	1023 (18.7)	1.67 (1.53–1.81, p < 0.001)
How often do you go to religious meetings or services	None/Little	7656 (52.6)	2994 (54.7)	-
	Often	3477 (23.9)	1204 (22.0)	0.89 (0.82–0.96, p = 0.002)
	I am not religious	3431 (23.6)	1280 (23.4)	0.95 (0.88–1.03, p = 0.230)
In your day to day life how often are you treated with less respect than other people	None/Little	10829 (74.4)	3325 (60.7)	-
	Often	1005 (6.9)	883 (16.1)	2.86 (2.59–3.16, p < 0.001)
	A few times a year	2730 (18.7)	1270 (23.2)	1.52 (1.40–1.64, p < 0.001)
How often are you treated with less respect than other people when you go to a doctor s office or other health care provider	None/Little	13358 (91.7)	4493 (82.0)	-
	Often	1206 (8.3)	985 (18.0)	2.43 (2.22–2.66, p < 0.001)
Per capita household income†	High	3260 (22.4)	609 (11.1)	-
	Low	2852 (19.6)	2044 (37.3)	3.84 (3.46–4.26, p < 0.001)
	Medium	8452 (58.0)	2825 (51.6)	1.79 (1.63–1.97, p < 0.001)
Household size	Two person household	7868 (54.0)	2336 (42.6)	-
	Single person household	2885 (19.8)	1379 (25.2)	1.61 (1.49–1.74, p < 0.001)
	Three and more person household	3811 (26.2)	1763 (32.2)	1.56 (1.45–1.68, p < 0.001)

† denotes variables selected for modeling of interactions (for intersectional analysis).

## Data Availability

The data underlying this article is available for registered users in All of Us platform. The code required to fetch the data and produce the results in this paper will be made available via the GitHub https://github.com/scherbakovdmitri/AllofUsDepressionDisparitiesLASSO/
